# Validating the early phototherapy prediction tool across cohorts

**DOI:** 10.3389/fped.2023.1229462

**Published:** 2023-10-09

**Authors:** Imant Daunhawer, Kai Schumacher, Anna Badura, Julia E. Vogt, Holger Michel, Sven Wellmann

**Affiliations:** ^1^ Department of Computer Science, ETH Zurich, Zurich, Switzerland; ^2^ Department of Neonatology, Hospital St. Hedwig of the Order of St. John, University Children’s Hospital Regensburg (KUNO), Regensburg, Germany

**Keywords:** machine learning, jaundice, children, baby, prediction

## Abstract

**Background:**

Hyperbilirubinemia of the newborn infant is a common disease worldwide. However, recognized early and treated appropriately, it typically remains innocuous. We recently developed an early phototherapy prediction tool (EPPT) by means of machine learning (ML) utilizing just one bilirubin measurement and few clinical variables. The aim of this study is to test applicability and performance of the EPPT on a new patient cohort from a different population.

**Materials and methods:**

This work is a retrospective study of prospectively recorded neonatal data from infants born in 2018 in an academic hospital, Regensburg, Germany, meeting the following inclusion criteria: born with 34 completed weeks of gestation or more, at least two total serum bilirubin (TSB) measurement prior to phototherapy. First, the original EPPT—an ensemble of a logistic regression and a random forest—was used in its freely accessible version and evaluated in terms of the area under the receiver operating characteristic curve (AUROC). Second, a new version of the EPPT model was re-trained on the data from the new cohort. Third, the predictive performance, variable importance, sensitivity and specificity were analyzed and compared across the original and re-trained models.

**Results:**

In total, 1,109 neonates were included with a median (IQR) gestational age of 38.4 (36.6–39.9) and a total of 3,940 bilirubin measurements prior to any phototherapy treatment, which was required in 154 neonates (13.9%). For the phototherapy treatment prediction, the original EPPT achieved a predictive performance of 84.6% AUROC on the new cohort. After re-training the model on a subset of the new dataset, 88.8% AUROC was achieved as evaluated by cross validation. The same five variables as for the original model were found to be most important for the prediction on the new cohort, namely gestational age at birth, birth weight, bilirubin to weight ratio, hours since birth, bilirubin value.

**Discussion:**

The individual risk for treatment requirement in neonatal hyperbilirubinemia is robustly predictable in different patient cohorts with a previously developed ML tool (EPPT) demanding just one TSB value and only four clinical parameters. Further prospective validation studies are needed to develop an effective and safe clinical decision support system.

## 1. Introduction

Hyperbilirubinemia of the newborn is one of the main diseases in neonatology (1) and the most common reason for readmission to hospital within the first month of life (2–4). The vast majority of newborn infants show visible jaundice within the first days of life (5). In most cases, spontaneous remission of this physiological jaundice occurs within two weeks (6). However, in a smaller but significant proportion of newborns, bilirubin levels increase to therapeutically relevant ranges, making phototherapy treatment necessary (5). If phototherapy is not applied in time, increased levels of bilirubin can lead to severe neurological sequelae and cause bilirubin-induced encephalopathy (BIND) (7). The individual suffering as well as costs to national health care systems can be enormous (8, 9).

More than two decades ago, Bhutani et al. developed percentiles to identify newborns at risk for severe hyperbilirubinemia (6). The so-called Bhutani nomograms introduced in 1999 assess the risk of developing severe hyperbilirubinemia based on postnatal age and bilirubin levels, providing a simple formula that remains an essential part of many national guidelines (5, 6, 10). Very recently the American Academy of Pediatrics updated the guideline on diagnosis and management of hyperbilirubinemia in the newborn infant to address also individual bilirubin dynamics (11).

However, BIND remains a major problem in countries with poor health care and even in industrialized countries cases of severe hyperbilirubinemia still occur (8, 9, 12). Over the last decades, inpatient stay of term and near-term born newborns after birth has been shortened to less than 48 h (13–15), while peak levels of bilirubin are only reached around the fifth day of life (7). The remaining appearance of BIND along with a shortened length of hospital stays after birth have increased the need for an early identification and precise surveillance of neonates at risk for significant hyperbilirubinemia.

Therefore, various studies investigated additional clinical risk factors—such as maternal, neonatal or birth-related factors—in order to develop refined models of risk stratification (5, 16–21). However, these models classify neonates into general risk groups that might not allow for a sufficiently personalized prediction of the actual clinical outcome for individual patients. In recent years, machine learning methods have become more important in the field of medicine (22, 23) and received increased attention also in the context of neonatal hyperbilirubinemia. For example, Castillo et al. (18) and Ferreira et al. (24) presented capable prediction models using different machine learning methods. However, both models only provide a single prediction within 24 h, which may not be convenient in daily clinical practice, where a prediction needs to be done after every follow-up measurement.

A promising and practical model was presented by Daunhawer et al. (25) using a combination of two different machine learning methods, namely a logistic regression and a random forest. The model requires only four variables that are always available: bilirubin value, gestational age, birth weight and postnatal age in hours. With these four variables, the model computes the probability of needed phototherapy within the next 48 h—a task for which the model achieved a strong predictive performance of 95.2% Area Under the Receiver Operating Characteristic curve (AUROC). The model was made publicly available as a web application called the “Early Phototherapy Prediction Tool” (EPPT) (26).

In this retrospective study, we apply the EPPT model from Daunhawer et al. (25) to a new cohort of healthy, term and near-term newborns ≥35 weeks of gestational age from the University Children's Hospital Regensburg (KUNO), Hospital St. Hedwig of the Order of St. John, University of Regensburg, Germany. First, we evaluate the model on the new cohort to test the external validity of the model under real-world distribution shift. Specifically, the new cohort has a distinct distribution of covariates (e.g., significantly fewer preterm cases) and slightly different phototherapy guidelines (see section 2.1). Second, we re-train the model on the data from the Regensburg cohort and based on the local guidelines for the administration of phototherapy. Hence, we validate the reliability of the EPPT in its original form and adapted to a new cohort to assess the prediction of therapeutically relevant hyperbilirubinemia. In particular, our study aims to test whether the model can be applied in everyday clinical practice to avoid cases of severe hyperbilirubinemia, to eventually reduce the frequency of blood sampling and ultimately reduce healthcare costs and improve patient outcomes.

## 2. Materials and methods

### 2.1. Study patients

We performed a retrospective study of prospectively recorded neonatal data of all infants born at the KUNO hospital, University of Regensburg, Germany, between January 1 2018 and December 31 2018. The study was approved by the ethics commission of the University of Regensburg (20-1945-104).

Inclusion criteria were: two or more total serum bilirubin (TSB) measurements prior to phototherapy treatment, gestational age with 34 completed weeks or more of gestation, absence of malformations requiring operation within the first month of life and absence of any genetic syndrome.

The following data were retrieved from the electronic medical records: gestational age at birth, birthweight, delivery mode (vaginal, elective caesarean section or secondary caesarean section), maternal Rh and blood group, TSB value and time elapsed since birth, if phototherapy was performed onset of first treatment. Phototherapy initiation limit was used according to the national guideline for hyperbilirubinemia diagnostic and treatment in neonates (AWMF guideline 024-007), which is based on the recommendations of the American Academy of Pediatrics (6). In detail, in the local KUNO guideline the phototherapy thresholds are increased by 1 mg/dl every 12 h, whereas the national guidelines increase it by 2 mg/dl every 24 h. All bilirubin measurements were performed as TSB using a Bilimeter 3D (Pfaff medical GmbH, Germany).

### 2.2. Dependent variable

Following previous work (25), we defined the outcome as the need of a phototherapy treatment within the next 48 h. We modeled the outcome as a binary variable (phototherapy vs. no phototherapy) that is positive if the patient's bilirubin value exceeded the guideline-specific critical value within the next 48 h and subsequently a phototherapy treatment was administered. As such, we assessed a neonate's risk of developing excessive bilirubin levels after *each* bilirubin measurement and not only at a fixed time point (e.g., 24 h after birth).

### 2.3. Machine learning

We used machine learning to predict the binary outcome given a small set of covariates that are generally available in most hospitals and outpatient settings. Specifically, we used the Early Phototherapy Prediction Tool [EPPT, (26)] in the same form it was designed and trained in the original study (25). In the following, we first provide a short description of the original model and its core components. Secondly, we explain how the model was re-trained on the new cohort.

### 2.4. Original EPPT

The EPPT is an ensemble of two models: a regularized logistic regression (LASSO) and a random forest classifier (27). As such, it combines a conventional statistical approach with a more modern machine learning method, which were shown to complement each other (25). The EPPT computes the probability of receiving a phototherapy treatment in the following 48 h based on four input variables: bilirubin value, gestational age, birth weight and postnatal age in hours. As such, the model requires only a handful of variables that are generally available and it can be used to obtain a prediction after each bilirubin measurement. Originally, the EPPT was trained on a dataset of 362 newborns of the University Children's Hospital Basel (UKBB). To assess the external validity of the model, we evaluated the original model without re-training it on the new cohort; specifically, we used the original model to compute the predictions for the new cohort and evaluated the predictions according to the local guidelines of the KUNO hospital.

### 2.5. Re-trained EPPT

Additionally, we developed a new version of the EPPT model that was trained on the data of the new cohort. We used the same model hyperparameter values as in the original study, namely a uniform weighting of both models in the ensemble, a regularization weight of 0.3 for the LASSO, and for the random forest we used 300 decision trees trained with Gini impurity as a split criterion, no depth limit, at least two samples per split, at least one sample per leaf, and random sampling of $\sqrt{k}$ variables in each tree, where *k* is the total number of input variables in the training data.

All analyses were performed using the Python programming language (version 3.6.3) and scikit-learn machine learning library (version 0.21.3).

### 2.6. Evaluation

To evaluate the performance of predicting the need of phototherapy, we followed previous work and used the AUROC, which considers the true positive rate and false positive rate at different decision thresholds. AUROC is a standard metric for the evaluation of binary classifiers and it allows us to draw a comparison to the results from previous work.

## 3. Results

### 3.1. KUNO dataset

The baseline characteristics of the neonates in the KUNO dataset are shown in Table 1. Overall, among 1,109 considered neonates fulfilling *a priori* inclusion and exclusion criteria, there were 154 cases (13.9%) that required a phototherapy treatment. As expected, neonates who received a phototherapy had a lower gestational age and birth weight on average compared to those who did not receive a phototherapy treatment. The median time of the first phototherapy was 77 h after birth. In total, there were 3,940 bilirubin measurements prior to the first respective phototherapy treatment with an average time of 35.3 h between measurements. Among these 3,940 observations, there were 239 (6.1%) positives, where a patient's bilirubin value exceeded the guideline-specific critical value within the next 48 h and a phototherapy treatment was administered.

**Table 1:** Baseline characteristics for all included neonates and neonates with phototherapy treatment respectively.

	All included neonates	Neonates with phototherapy
	*n* = 1,109	*n* = 154
Gestational age, in weeks	38.4 (36.6–39.9)	35.4 (33.7–37.1)
Birth weight, in kg	3.1 (2.6–3.6)	2.5 (2.0–3.0)
Delivery mode		
Spontaneous	496 (44.7)	102 (66.2)
Caesarean Section	592 (53.4)	51 (33.1)
First bilirubin value, in mg/dl	7.0 (2.2–10.4)	2.8 (2.0–11.2)
Time point of first phototherapy, in hours since birth	N/A	77.0 (51.7–94.3)

For categorical variables, we report the counts and relative frequencies in parentheses. For continuous variables, we report the median values and 25% and 75% percentiles in parentheses.

### 3.2. Predictive performance

**Figure 1:**
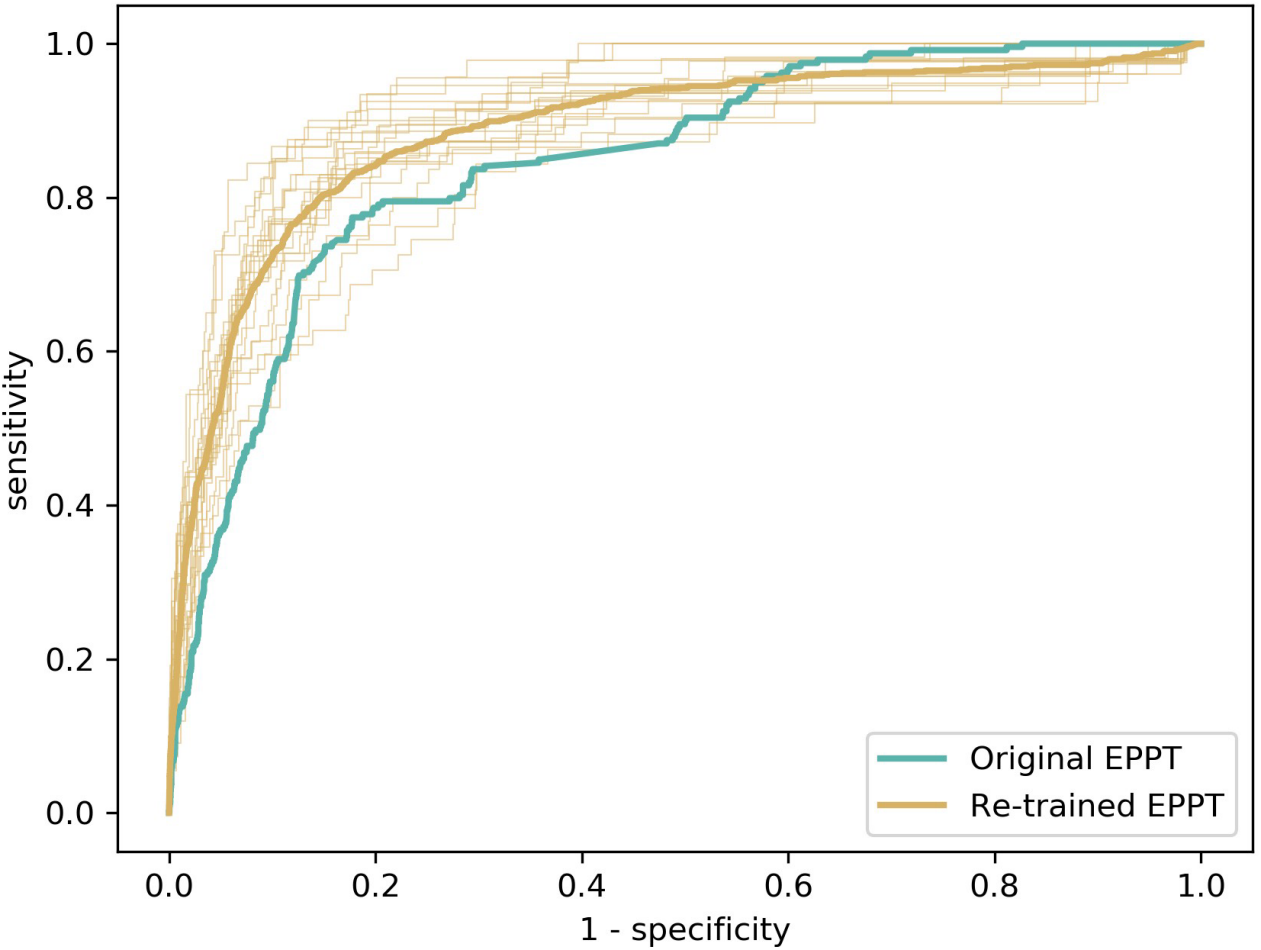
Evaluation of the predictive performance of the EPPT on the KUNO dataset. We compare the original EPPT trained on the UKBB dataset with the same model trained on the KUNO dataset. The bold lines show the operator characteristic (ROC) curve for the respective models. The fine lines show the ROC curve for the individual cross-validation folds of the re-trained model.

Following previous work (25), we evaluated the predictive performance of the EPPT in terms of the AUROC. The results are presented in Figure 1. First, we employed the EPPT in its original form, i.e., without re-training the model on the KUNO dataset. Consequently, we used the complete KUNO dataset to evaluate the performance of the model. We observed that the original EPPT achieves a predictive performance of 84.6% AUROC, which is about 11 percentage points lower than in the original study which evaluated the model on the UKBB dataset. Second, we re-trained the EPPT model on part of the KUNO dataset and evaluated the model on a holdout set. Specifically, we used a 20-fold cross validation, stratified by patient such that no individual occurs in both the training and test set. We computed the average performance and standard deviation across folds and observed that the re-trained EPPT achieves a predictive performance of 88.8% (± 3.0%) AUROC.

To validate the robustness of our results despite the limited sample size, we performed an additional analysis, where we trained the model on a subset of the data. We observed that even with 25% of the data, the model achieved a predictive performance within the range of our previous results (Table 2).

**Table 2:** Ablation study using a subset of the training data.

Training dataset size	AUROC	Sensitivity	Specificity	PPV
25%	86.325 (± 2.9)	9.663 (± 5.6)	99.616 (± 0.3)	63.483 (± 26.8)
50%	87.965 (± 3.3)	14.962 (± 5.6)	99.374 (± 0.4)	62.781 (± 14.4)
75%	88.365 (± 3.3)	17.015 (± 5.7)	99.315 (± 0.4)	62.221 (± 13.262)
100%	88.761 (± 3.0)	18.514 (± 5.5)	99.266 (± 0.4)	63.431 (± 12.7)

In each row, we report the AUROC, sensitivity, specificity, and positive predictive value (PPV) for the EPPT trained using a random subset of the training data. Each value denotes the mean (in percent) computed across 20 cross-validation folds and in parentheses we show the standard deviation.

### 3.3. Variable importance

**Figure 2:**
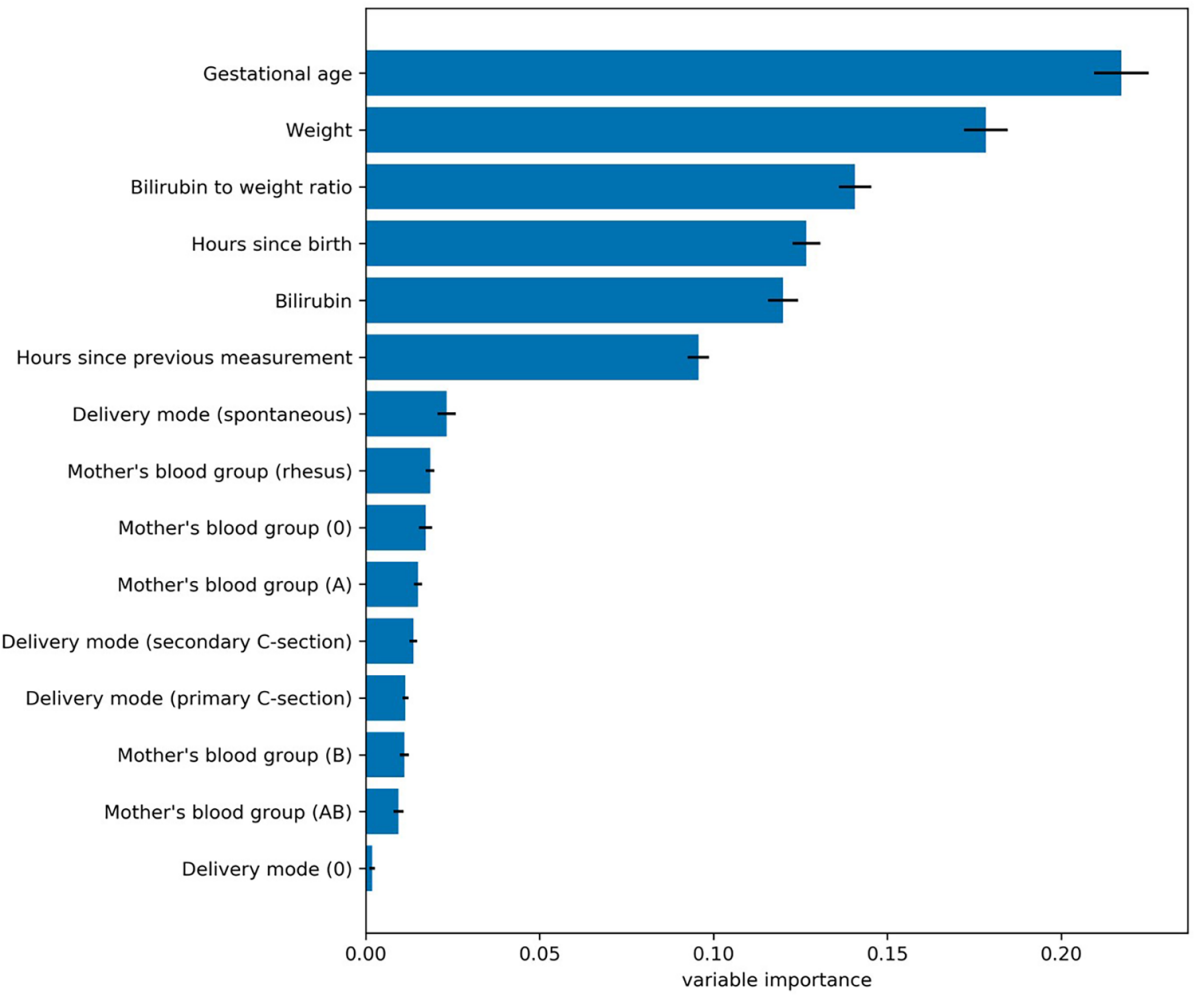
Variable importance values for the random forest trained using all variables in the dataset. Each bar denotes the relative importance of the respective variable and the standard deviation across cross validation folds is shown with error bars. The results indicate that the predictive performance of the random forest depends mostly on a subset of five variables, which correspond to the same variables used by the original EPPT.

**Figure 3:**
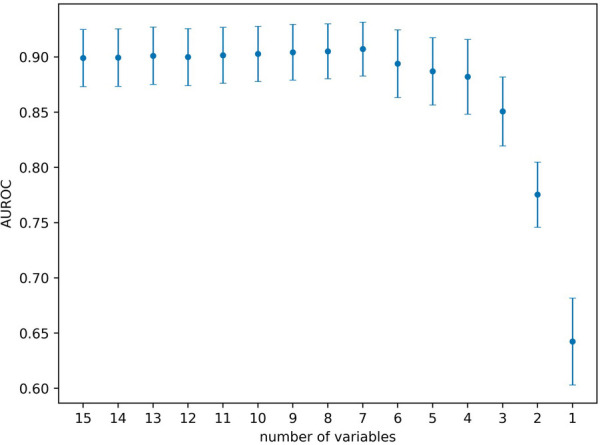
Backwards feature selection using the random forest. Starting with all variables, in each step we remove the variable with lowest variable importance. Markers denote the average AUROC across 20 cross-validation folds and error bars denote the standard deviation respectively. The last six variables removed were the following (in the given order): hours since previous measurement, bilirubin, hours since birth, gestational age, birth weight, bilirubin to weight ratio.

To estimate the effect the individual variables have on the prediction, we estimated their relevance (i.e., variable or feature importance) in the random forest. Figure 2 presents the results for the random forest trained using all variables in the dataset. The results indicate that the predictive performance depends on only a handful of variables. The five most important variables (gestational age, birth weight, bilirubin to weight ratio, hours since birth, bilirubin value) correspond to the same variables used in the original EPPT, which confirms the utility of these variables for the phototherapy treatment prediction across datasets. In Figure 3, we provide additional results for which we performed an iterative backwards variable selection, showing that the bilirubin to weight ratio is the last variable to be removed, which is in line with the original study.

### 3.4. Sensitivity and specificity analysis

**Figure 4:**
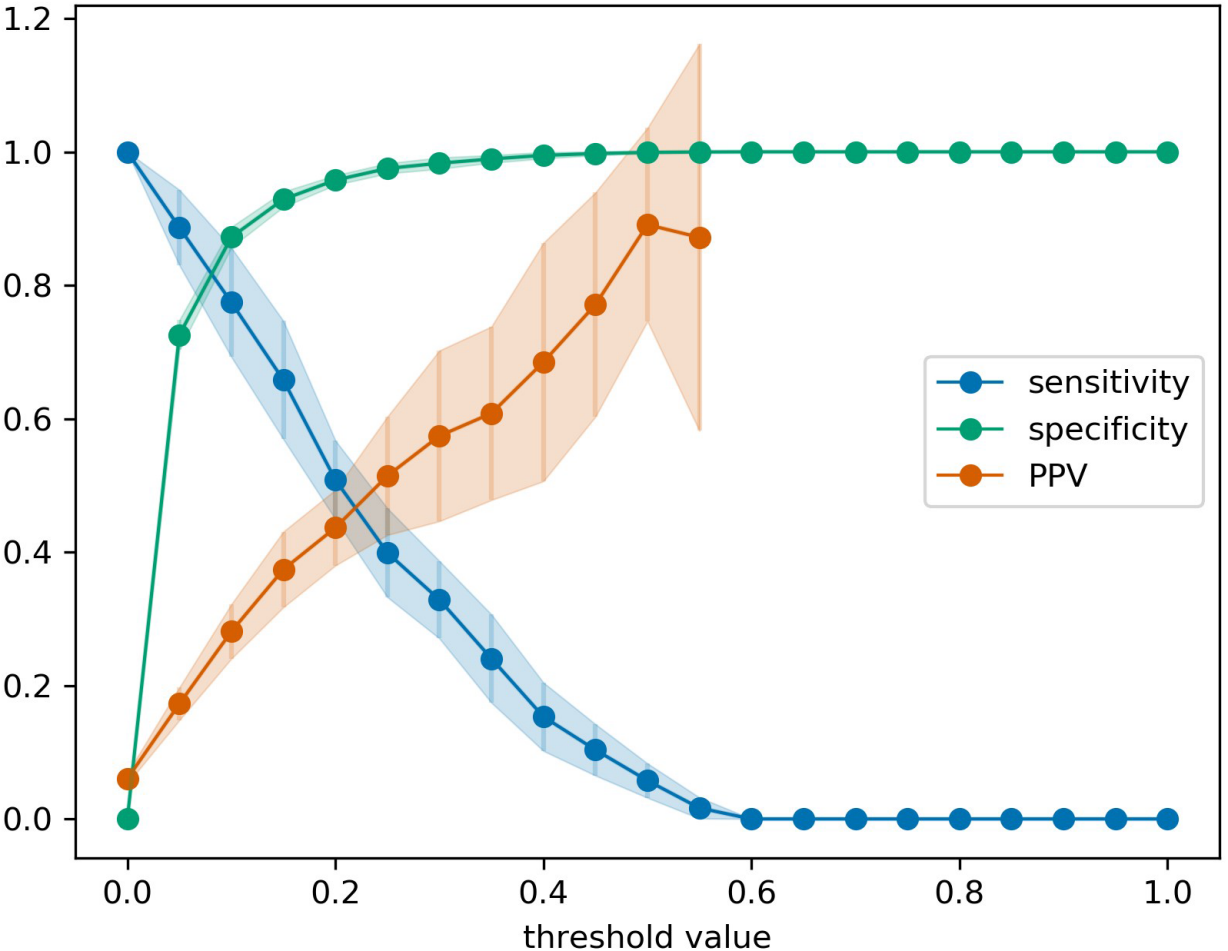
Sensitivity, specificity, and positive predictive value (PPV) for the re-trained EPPT as a function of the decision threshold value. Error bars denote the standard deviation across 20 cross-validation folds. PPV values for threshold values larger than 0.575 are missing, because there were individual folds without positive predictions as the predicted probabilities did not exceed the threshold value.

While the AUROC metric provides a comprehensive assessment of the predictive performance of a model, the value is not easy to interpret in terms of the practical utility of the model. Therefore, we also evaluated the confusion matrices on a measurement- and patient-level. On the level of individual bilirubin measurements, the EPPT (i.e., the original model without any re-training) achieves a sensitivity of 33.8% and a specificity of 95.9% on the new population. In absolute numbers, the model correctly predicted 77 out of 239 phototherapies within 48 h, but also made 151 false positive and 162 false negative predictions based on a total of 3,940 predictions (one after each bilirubin measurement). On the level of individual patients, the model achieves a sensitivity of 39.6% and a specificity of 90.7% and in absolute numbers correctly predicted 61 out of 154 phototherapy cases, but also made 89 false positive and 93 false negative predictions based on 1,109 patients in total. Additionally, for the re-trained EPPT, in Figure 4 we provide a refined analysis of the sensitivity, specificity, and positive predictive value as a function of the decision threshold value. The results indicate that a threshold value between 0.2–0.3 provides a good balance of sensitivity and positive predictive value (PPV), as well as a high specificity. The value is slightly lower than the decision threshold of 0.38 suggested in the original study.

## 4. Discussion

In this study, we demonstrated that the considered model (i.e., EPPT), consisting of a logistic regression and a random forest, can be successfully applied to a different population of newborn infants. The model was able to predict the need of phototherapy treatment 48 h in advance with a predictive performance of 84.6% AUROC in its original form and 88.8% AUROC when trained on the new cohort. Thus, we present additional evidence that machine learning methods can be used for enhancing the early detection of clinically significant hyperbilirubinemia.

One strength of this study is that it is based on a large dataset of newborn infants. Additionally, we found that the four variables (weight, gestational age, bilirubin and time since birth) yield a high predictive performance that is not significantly improved when we consider additional variables, which confirms the results from previous work (25). This highlights the general importance of these factors for the prediction of hyperbilirubinemia and emphasizes the universality of the developed model.

EPPT shows great potential in its practicability in the clinical context. It can provide a risk prediction after every follow-up measurement, not only at a certain time point like in previous mentioned studies of Castillo et al. (18) or Ferreira et al. (24). The number of variables required for the prediction is small and should be available in most hospitals, in outpatient-settings, or even in case of homebirth as long as a bilirubin measurement is possible.

While bilirubin nomograms used in many national guidelines only classify neonates into general risk groups, the EPPT can supply a precise individual probability, which may illustrate the individual risk more clearly to caregivers. Compliance is an essential condition of successful follow-up programs (28–30). Applying the EPPT in daily routine may contribute to a better compliance in follow-up programs, since the model is easily available (online or via mobile App), cost-efficient, simple to use and delivers specific, understandable instructions for all caregivers. Lastly, applying EPPT in daily practice may minimize the need of blood sampling since bilirubin measurements might be stopped earlier, which can be an interesting opportunity for future work.

And finally, the EPPT works with just one bilirubin measurement and does not require at least two bilirubin measurements as our previously published pharmacometrics-based mathematical-statistical computer program (PMX-based algorithm) (31).

The model's performance in our cohort was lower with 84.6% AUROC and after retraining with 88.8% compared to the value of 95.2% reported in the original study (25). However, the performance lies in a similar range and it is comparable to the results achieved by alternative models (16, 18, 19, 24) for which there are no existing studies of their external validity. The lower performance can be explained by differences between local guidelines and included cohorts. For example, in our cohort the proportion of preterm newborns was significantly lower as we only included neonates ≥35 weeks of gestational age. Additionally, the thresholds for phototherapy treatment in the considered cohort are slightly different since they are interpolated compared to those in the AAP and UKBB guidelines. This may contribute to a lower incidence of phototherapy treatment in our study and an ambiguous labelling of a few predictions that might be labelled as false positives based on the local guidelines but as true positives according to the guidelines of the original study. Hence, the lower performance can be partly explained by the distribution shift of covariates and outcome variables across datasets, and partly by the inherent difficulty of the new cohort for which even a model that was trained on a subset of the new cohort achieves a performance of only 88.8% AUROC on a holdout set.

Before EPPT can be implemented into clinical decision-making, a prospective cohort study is needed to test its sensitivity and specificity. Furthermore, one needs to define a specific cut-off which determines the optimal timing for stopping follow-up measurements of bilirubin. To achieve this the clinical study could be divided into various arms with different cut-offs. Albeit our study dataset contained only TSB values, which are not affected by various skin colours, and race has been removed in the latest AAP guideline on neonatal hyperbilirubinemia (11), we will investigate in the future various populations with different skin colours which may improve the predictive performance of the EPPT and may lead to a better generalization. To further corroborate the validity of the model in more individualized settings, future studies should include higher numbers of preterm neonates as well as an integration of local guidelines for phototherapy treatment.

Our study suggests several opportunities for future research. For example, it is of clinical interest, to predict not only the necessity of a phototherapy treatment, but also its duration, intensity, or risk for a rebound hyperbilirubinemia. Moreover, the influence of maternal medication during pregnancy could be examined as a potential risk factor for subsequent significant hyperbilirubinemia.

## Data Availability

The raw data supporting the conclusions of this article will be made available by the authors, without undue reservation.
